# A Conjugative 38 kB Plasmid Is Present in Multiple Subspecies of *Xylella fastidiosa*

**DOI:** 10.1371/journal.pone.0052131

**Published:** 2012-12-14

**Authors:** Elizabeth E. Rogers, Drake C. Stenger

**Affiliations:** United States Department of Agriculture, Agricultural Research Service, Parlier, California, United States of America; University of Wisconsin-Milwaukee, United States of America

## Abstract

A ∼38kB plasmid (pXF-RIV5) was present in the Riv5 strain of *Xylella fastidiosa* subsp. *multiplex* isolated from ornamental plum in southern California. The complete nucleotide sequence of pXF-RIV5 is almost identical to that of pXFAS01 from *X. fastidiosa* subsp. *fastidiosa* strain M23; the two plasmids vary at only 6 nucleotide positions. BLAST searches and phylogenetic analyses indicate pXF-RIV5 and pXFAS01 share some similarity to chromosomal and plasmid (pXF51) sequences of *X. fastidiosa* subsp. *pauca* strain 9a5c and more distant similarity to plasmids from a wide variety of bacteria. Both pXF-RIV5 and pXFAS01 encode homologues of a complete Type IV secretion system involved in conjugation and DNA transfer among bacteria. Mating pair formation proteins (Trb) from *Yersinia pseudotuberculosis* IP31758 are the mostly closely related non-*X. fastidiosa* proteins to most of the Trb proteins encoded by pXF-RIV5 and pXFAS01. Unlike many bacterial conjugative plasmids, pXF-RIV5 and pXFAS01 do not carry homologues of known accessory modules that confer selective advantage on host bacteria. However, both plasmids encode seven hypothetical proteins of unknown function and possess a small transposon-associated region encoding a putative transposase and associated factor. Vegetative replication of pXF-RIV5 and pXFAS01 appears to be under control of RepA protein and both plasmids have an origin of DNA replication (*oriV*) similar to that of pRP4 and pR751 from *Escherichia coli*. In contrast, conjugative plasmids commonly encode TrfA and have an *oriV* similar to those found in IncP-1 incompatibility group plasmids. The presence of nearly identical plasmids in single strains from two distinct subspecies of *X. fastidiosa* is indicative of recent horizontal transfer, probably subsequent to the introduction of subspecies *fastidiosa* to the United States in the late 19^th^ century.

## Introduction

Horizontal gene transfer plays a critical role in bacterial adaptation and evolution. On average, 81% of the genes in a typical bacterial genome have been involved in a horizontal transfer event at some point in the past [1]. One of the most common mechanisms for DNA exchange is via conjugative plasmids that encode type IV secretion systems (T4SS), a broad class of macromolecular translocation machinery. There are three main types of T4SS: i) conjugation systems that transfer DNA and, in some instances, DNA-binding proteins; ii) effector translocator systems that deliver proteins and other effectors to eukaryotic cells during bacterial infection of eukaryotic hosts; and iii) DNA uptake or release systems that move DNA between the interior of the cell and the extracellular environment [2]. Conjugation systems are found both on self-transmissible or conjugative plasmids, circular DNA molecules that replicate independently of the bacterial chromosome, and on integrative and conjugative elements that integrate into the chromosome, excising and forming a circular intermediate prior to translocation [2]. One well characterized conjugation system is VirB/D4 from *Agrobacterium tumefaciens,* which is composed of a cell-envelope spanning secretion channel and an extracellular pilus that contacts the recipient cell [3]. For VirB/D4, the translocation system consists of VirB2-11 proteins forming the secretion channel, the VirD4 substrate receptor or type IV coupling protein (T4CP), and proteins for pilus formation and DNA substrate processing [2]. Many conjugative plasmids also contain accessory modules encoding cargo proteins which act as virulence factors, confer resistance to antibiotics/heavy metals, or catabolize toxic organic substances [4].

*Xylella fastidiosa* is a fastidious, xylem-limited Gram-negative bacterial phytopathogen causing numerous vascular occlusion and water stress diseases including Pierce’s disease of grape, almond leaf scorch, oleander leaf scorch, and other diseases of perennial crops and landscape plants [5]. Four subspecies of *X. fastidiosa* have been identified based on a multi-locus sequence typing (MLST) phylogeny [6], [7]. Subspecies *fastidiosa* contains strains of low genetic diversity that cause Pierce’s disease and sometimes almond leaf scorch in the U. S. and diverse strains from Central America; subsp. *fastidiosa* is thought to have been introduced to the U. S. in the late 19^th^ century [8]. Subspecies *multiplex* is an endemic North American clade capable of infecting numerous hosts (but generally not grapevine) [9]–[11]. Subspecies *pauca* contains South American strains causing citrus variegated chlorosis and coffee leaf scorch [12]. Subspecies *sandyi* consists of closely related strains isolated from oleander in California and Texas and is thought to have been introduced to the United States approximately 30 years ago [6]. Currently, 5 fully sequenced and annotated *X. fastidiosa* genomes are available (*pauca* strain 9a5c [13], *fastidiosa* strain Temecula [14], *multiplex* strain M12 [15], *fastidiosa* strain M23 [15], and *fastidiosa* strain GB514 [16]). Two additional sequences (*multiplex* strain Dixon, GenBank accession number NZ_AAAL00000000.2, and *sandyi* strain Ann-1, GenBank accession number NZ_AAAM00000000.3) are incomplete, unassembled shotgun sequences; the Ann-1 sequence may have been derived from a mixed culture and appears to be contaminated with sequences from a *multiplex* strain [17].

Here, we characterize two closely related 38kB conjugative plasmids of *X. fastidiosa*. Plasmid pXF-RIV5 was isolated from the Riv5 strain of *X. fastidiosa* subspecies *multiplex* [18]; complete sequence of pXF-RIV5 was determined in this current work. Plasmid pXFAS01 is known only as a circular contig discovered during the complete genome sequencing of *X. fastidiosa* subspecies *fastidiosa* strain M23 [15]. While minimal annotation of pXFAS01 accompanies the sequence in GenBank, no analysis of the type IV secretion system, origin of transfer or origin of replication from pXFAS01 has been presented previously. This work presents analyses of gene complement and phylogeny of these two closely related plasmids to reveal i) an evolutionary history of recombination among divergent sources to generate the mosaic backbone shared by pXF-RIV5 and pXFAS01, and ii) evidence of recent translocation of plasmid DNA via conjugation among distinct subspecies of *X. fastidiosa*.

## Materials and Methods

### Culture and MLST of *X. fastidiosa* subspecies *multiplex* strain Riv5

Isolation of *X. fastidiosa* strain Riv5 from ornamental plum (*Prunus cerasifera*) was described previously [18]. Strain Riv5 cultures were grown in liquid periwinkle wilt (PW) medium for 7–10 days at 28C and used to inoculate plates containing solid PW medium. After 7–10 days of growth at 28C, bacterial colonies were washed from 10 PW plates and extracted for total genomic DNA [11]. Genomic DNA was used as template for PCR amplification of seven housekeeping genes (*cysG, gltT, holC, malF, leuA, nuoL, petC*) and *pilU* [7]; consensus sequences were determined for each amplified region based on sequences of three independent clones per PCR product. Consensus sequences for each amplified region were concatenated into a single sequence. MLST was performed as described [7] with concatenated Riv5 sequences aligned with the corresponding concatenated sequences from multiple strains representative of each *X. fastidiosa* subspecies available in GenBank using CLUSTALX. Phylogenetic placement of strain Riv5 was determined based on a neighbor-joining tree (1000 bootstrap replications) using the multiple alignment of concatenated MLST sequences as input data.

### Plasmid DNA isolation and sequencing

Previously, strain Riv5 was shown to harbor a large plasmid (designated here as pXF-RIV5), yielding multiple products when digested with *Hin*dIII [18]. Purification of pXF-RIV5 DNA was as described [18] from Riv5 cultures grown under the same regime as that used to extract genomic DNA. Purified plasmid DNA was digested with *Hin*dIII; each resulting fragment was gel purified and ligated into *Hin*dIII digested pGEM7Zf+ (Promega, Madison, WI). Ligation products were transformed into *Escherichia coli* JM109; recombinant plasmids bearing *Hin*dIII inserts of pXF-RIV5 were sequenced using a combination of universal (M13 forward and reverse) and custom primers. As preliminary sequences of insert termini obtained with universal primers indicated a very close relationship with pXFAS01, custom primers were based on the known sequence of pXFAS01 (GenBank Accession NC_010579.1) associated with the *X. fastidiosa* subspecies *fastidiosa* strain M23 genome sequence [15]. The complete nucleotide sequence of pXF-RIV5 (GenBank Accession JX548317) was assembled using the sequence of pXFAS01 as a scaffold.

### DNA sequence analysis and annotations

Open reading frames were identified based on similarity to pXFAS01 and using ORF Finder (http://www.ncbi.nlm.nih.gov/gorf/gorf.html). Annotations are derived from similar sequences identified using BLAST [19] and NCBI conserved domain searches [20]. The origin of conjugative transfer (*oriT*) was identified by similarity to the *oriT* regions of pRP4 and pR751 [21]. The tandem repeats in *oriV* were found using Repfind (http://zlab.bu.edu/repfind/index.shtml). A map of pXF-RIV5 was drawn using GENtle (http://gentle.magnusmanske.de/) open software package.

### Phylogenetic analysis of pXF-RIV5

Phylogeny of three proteins encoded by pXF-RIV5, representing three distinct genetic modules resident on pXF-RIV5 (and pXFAS01), were selected for examination. RepA represents a protein involved in DNA replication; TraI is a relaxase homologue representing the *tra* module (conjugative transfer functions); and TrbG is predicted to be an outer membrane protein and represents the *trb* module (mating pair formation functions).

Taxa selected for inclusion were based on results of BLAST P searches of the GenBank nonredundant protein database using the corresponding homologues of RepA, TraI and TrbG encoded by pXF-RIV5 as queries. A neighbor-joining tree (1000 bootstrap replicates) for each protein was constructed based on a multiple alignment of amino acid sequences generated using CLUSTALX. Nodes bearing <70% bootstrap support were considered unreliable and collapsed to polytomies.

## Results

### Riv5 is a strain of subspecies *multiplex*

Genomic DNA sequences used for MLST of strain Riv5 were deposited as GenBank Accessions JX679700-JX67907. Phylogeny of concatenated sequences of the eight genes examined by MLST indicated that strain Riv5 clustered with *X. fastidiosa* strains of subspecies *multiplex,* including the fully-sequenced *multiplex* strain M12 (data not shown). Phylogeny of each individual gene used for MLST also clustered strain Riv5 with strains of subspecies *multiplex* (data not shown). These results are consistent with phylogenetic placement of strain Riv5 based on 16S-23S rRNA spacer sequences [18].

### pXF-RIV5 contains a type IV secretion system

**Figure 1:**
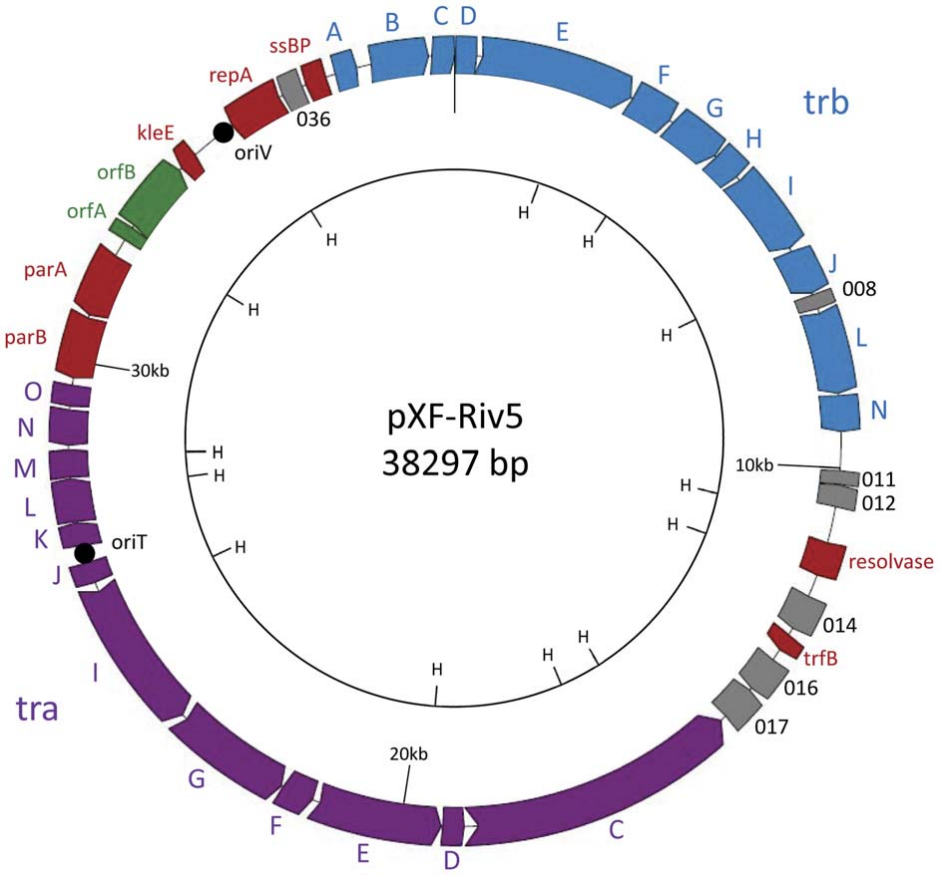
Genetic map of pXF-RIV5. The open reading frames are colored according to presumed function: red, plasmid replication and partition; purple, conjugative transfer (tra); blue, mating pair formation (trb); green, transposon associated; gray, hypothetical proteins of unknown function. The origin of replication (*oriV*) and the origin of transfer (*oriT*) are indicated by black circles. An inner circle marks HindIII restriction sites (H) used in subcloning.

The complete nucleotide sequence of pXF-RIV5 is 38,297 bp in length with a G+C content of 49.2%, which is similar to the 51–52% G+C content of sequenced *X. fastidiosa* genomes [13], [15]. As detailed in Table 1 and Figure 1, pXF-RIV5 has 33 ORFs encoding proteins similar to characterized proteins of known function from other organisms while seven ORFs encode hypothetical proteins for which functions of homologues identified in GenBank are unknown. The two largest groups of genes are the conjugative transfer (*tra*) and mating pair formation (*trb*) modules. Together, these two genetic modules encode homologues of all proteins necessary for a functional T4SS [2]. Genes for plasmid replication (*repA*, *kleE*, and *ssBP*) and partition (*parA* and *parB*) also are present. Two genes, *orfA* and *orfB*, encode proteins similar to transposon-associated recombinase and transposase, respectively; no other transposon-like elements on pXF-RIV5 were identified. Unlike many conjugative plasmids from animal pathogens or environmental samples, no accessory modules containing homologues of known virulence factors, antibiotic/heavy metal resistance, or catabolism of toxic organic compounds were encoded by pXF-RIV5.

**Table 1:** Annotation of open reading frames from pXF-RIV5.

Name	start	stop	# aa	strand	product	most closely related gene product	% aa identity
TrbD	3	323	107	+	conjugal transfer protein, ATPase, VirB3 family	conjugation protein TrbD [Azoarcus sp. EbN1] YP_195564.1	71
TrbE	311	2875	855	+	conjugal transfer protein, ABC transporter-like, VirB4 family	conjugal transfer protein TrbE [Yersinia pseudotuberculosis IP 31758] YP_001393245.1	69
TrbF	2872	3588	239	+	conjugal transfer protein, inner membrane protein, VirB8 family	conjugal transfer protein TrbF [Yersinia pseudotuberculosis IP 31758] YP_001393246.1	61
TrbG	3605	4495	297	+	conjugal transfer protein, periplasmic or outer membrane protein, VirB9 family	conjugal transfer protein TrbG [Yersinia pseudotuberculosis IP 31758] YP_001393247.1	68
TrbH	4498	4974	159	+	conjugal transfer protein, putative lipoprotein, VirB7 family	conjugal transfer protein TrbH [Yersinia pseudotuberculosis IP 31758] YP_001393248.1	41
TrbI	4980	6380	467	+	conjugal transfer protein, inner membrane protein, VirB10 family	conjugal transfer protein TrbI [Yersinia pseudotuberculosis IP 31758] YP_001393249.1	53
TrbJ	6399	7172	258	+	conjugal transfer protein, periplasmic or outer membrane protein, VirB5 family	conjugative transfer protein TrbJ [Burkholderia pseudomallei Pakistan 9] ZP_03794994.1	66
pRiv5_008	7189	7425	79	+	hypothetical protein, putative lipoprotein attachment site	lipoprotein [Aggregatibacter actinomycetemcomitans D11S-1] YP_003966129.1	44
TrbL	7447	8817	457	+	conjugal transfer protein, inner membrane protein, VirB6 family	conjugal transfer protein TrbL/VirB6 [Yersinia pseudotuberculosis IP 31758] YP_001393251.1	54
TrbN	8823	9422	200	+	conjugal transfer protein, lytic transglycosylase domain, possible VirB1	conjugal transfer protein TrbN [Yersinia pseudotuberculosis IP 31758] YP_001393252.1	63
pRiv5_011	10035	10262	76	-	conserved hypothetical protein, Pfam06156/DUF972 family	hypothetical protein XMIN_4535 [Xanthomonas citri pv. mangiferaeindicae LMG 941] ZP_09883050.1	37
pRiv5_012	10259	10627	123	-	hypothetical protein	hypothetical protein EGYY_28500 [Eggerthella sp. YY7918] YP_004712228.1	44
resolvase	11170	11733	188	+	site-specific serine recombinase family protein	putative resolvase [Methylomicrobium alcaliphilum] YP_004901765.1	76
pRiv5_014	12064	12657	198	+	hypothetical protein	hypothetical protein MYA_6037 [Burkholderia sp. KJ006] YP_006337102.1	43
TrfB	12725	13039	105	+	probable TrfB transcriptional repressor protein	hypothetical protein BBR47_02790 [Brevibacillus brevis NBRC 100599] YP_002769760.1	61
pRiv5_016	13216	13809	198	+	hypothetical protein	hypothetical protein MYA_6037 [Burkholderia sp. KJ006] YP_006337102.1	54
pRiv5_017	13850	14437	196	-	hypothetical protein	hypothetical protein Acife_3030 [Acidithiobacillus ferrivorans SS3] YP_004785429.1	55
TraC	14505	18992	1496	-	conjugal transfer protein, topoisomerase/primase-like	TraC DNA primase [Plasmid QKH54] YP_619864.1	40
TraD	18998	19360	121	-	conjugal transfer protein, inner membrane protein	TraD protein [IncP-1 plasmid pKJK5] YP_709180.1	49
TraE	19363	21420	686	-	conjugal transfer protein, topoisomerase-primase domain	TraE [Pseudomonas putida] YP_003162628.1	73
TraF	21449	21985	179	-	conjugal transfer protein, peptidase/pilin processing protease	TraF protein of DNA transfer system [Methylophaga sp. JAM7] YP_006297569.1	68
TraG	21982	23916	645	-	conjugal transfer protein, coupling protein, VirD4 family	conjugal transfer protein TraG [Yersinia pseudotuberculosis IP 31758] YP_001393286.1	79
TraI	23913	26405	831	-	conjugal transfer protein, relaxase/mobilization domain	conjugal transfer protein TraI [Yersinia pseudotuberculosis IP 31758] YP_001393287.1	45
TraJ	26440	26793	118	-	conjugal transfer protein, relaxosome component	conjugal transfer relaxosome component TraJ [Aeromonas caviae Ae398] ZP_08522309.1	54
TraK	27018	27413	132	+	conjugal transfer protein, putative oriT binding protein	TraK protein [IncP-1 plasmid pKJK5] YP_709187.1	47
TraL	27413	28138	242	+	conjugal transfer protein, contains P-loop nucleotide binding domain	TraL protein [Pseudomonas sp. ADP] NP_862455.1	71
TraM	28138	28590	151	+	conjugal transfer protein, transcriptional activator	traM gene product [Methylomicrobium alcaliphilum] YP_004901800.1	54
TraN	28652	29248	199	-	conjugal transfer protein, mating pair stabilization protein	hypothetical protein pKJK5_51 [IncP-1 plasmid pKJK5] YP_709190.1	48
TraO	29275	29628	118	-	conjugal transfer protein, putative membrane protein	putative conjugation protein TraO [Azoacrus sp. EbN1] YP_195664.1	53
parB-like	29726	30757	344	-	contains parB-like nuclease domain, putative partition site DNA binding protein	ParB equivalent nuclease [uncultured bacterium] YP_112421.1	71
parA-like	30754	31833	360	-	putative ATPase involved in plasmid replication and partition	IncC1 protein [uncultured bacterium] NP_598102.1	62
orfA	32062	32286	75	+	orfA family, site-specific serine recombinase, transposon-assoc.	transposon IS605 OrfA [Methylacidiphilum infernorum V4] YP_001941027.1	85
orfB	32280	33476	399	+	orfB family, helix-turn-helix domain, probable transposase	transposon IS605 OrfB [Methylacidiphilum infernorum V4] YP_001941028.1	72
KleE	33517	33837	107	-	probable KleE stable plasmid inheritance protein	KleE protein [Plasmid pB3] YP_133959.1	46
RepA	34560	35471	304	-	protein involved in plasmid replication, exact role unknown	RepA [Acidithiobacillus caldus SM-1] YP_004750509.1	75
pRiv5-036	35493	35849	119	-	hypothetical protein	hypothetical protein pSB102_p07 [Plasmid pSB102] NP_361021.1	54
ssBP	35893	36252	120	-	single-strand DNA binding protein	single-strand DNA-binding protein [uncultured bacterium] YP_112367.1	61
TrbA	36370	36726	119	+	conjugal transfer protein, helix-turn-helix containing tx regulator	conjugal transfer protein TrbA [Yersinia pseudotuberculosis IP 31758] YP_001393241.1	73
TrbB	36930	37892	321	+	conjugal transfer protein, ATPase, VirB 11 family	conjugal transfer protein TrbB [Yersinia pseudotuberculosis IP 31758] YP_001393242.1	70
TrbC	37905	38297	131	+	conjugal transfer protein, subunit of bacterial pilus, VirB2 family	conjugal transfer protein TrbC [Yersinia pseudotuberculosis IP 31758] YP_001393243.1	71

The most closely related gene product was identified using BLAST P and excludes other proteins from *X. fastidiosa*.

### Relationship of pXF-RIV5 to other *X. fastidiosa* plasmids

The sequence of pXF-RIV5 is almost identical to pXFAS01 from *X. fastidiosa* subsp. *fastidiosa* strain M23 [15]. For consistency, nucleotide coordinates of pXF-RIV5 were assigned to correspond to nucleotide coordinates designated for pXFAS01. Alignment of pXF-RIV5 and pXFAS01 revealed polymorphism, all of which are transitions, at only six nucleotide positions over the entire ∼38 kB length. Two transitions are located in intergenic regions at nt 9,556 (between *trbN* and ORF11) and at nt 36,281 (between *ssBP* and *trbA*). The other four transitions are located in the *traI* gene encoding a conjugative relaxase homologue. Two transitions (nts 24,789 and 24,921) in the *traI* gene were synonymous substitutions that did not alter predicted protein sequence. The remaining two transitions in the *traI* gene were nonsynonymous substitutions that altered the codon for amino acid 462 (nt 25,019) from proline (pXFAS01) to serine (pXF-RIV5) or altered the codon for amino acid 386 (nt 25,250) from serine (pXFAS01) to proline (pXF-RIV5). Although both nonsynonymous substitutions are not in highly conserved portions of TraI, potential alteration of function cannot be excluded. Clustering of four out of six polymorphic sites in less than 500 bp of a 38 kB plasmid raises the possibility that all substitutions in *traI* were introduced by a single recombination event between pXF-RIV5 or pXFAS01 and a closely related plasmid.

The plasmid (pXF51) of *X. fastidiosa* subsp. *pauca* strain 9a5c encodes a partial *trb* module [22] which is 96% identical at the nucleotide level over almost 9 kB of pXF-RIV5 and pXFAS01, spanning *trbE* through *trbN* (nts 481 – nts 9401). Strain 9a5c also has an extensive cluster of *trb* genes on the chromosome [22] sharing 97% nucleotide sequence identity with ∼10 kB of pXF-RIV5 and pXFAS01 (nts 37612 – nts 9417). A small region of pXF-RIV5 and pXFAS01 (140 bp; nts 9276 – nts 9414) shares 89% nucleotide sequence identity to 25 kB IncP-1 plasmids (pXF-RIV11, pXF-RIV16, pXF-RIV19, and pXF-RIV25) from mulberry-infecting strains of *X. fastidiosa* [18]. In pXF-RIV5 and pXFAS01, the homologous region is within the *trbN* gene; however, in the IncP-1 plasmids, the complete *trbN* gene is not present.

### DNA replication elements and T4SS components of pXF-RIV5 have distinct evolutionary histories

**Figure 2:**
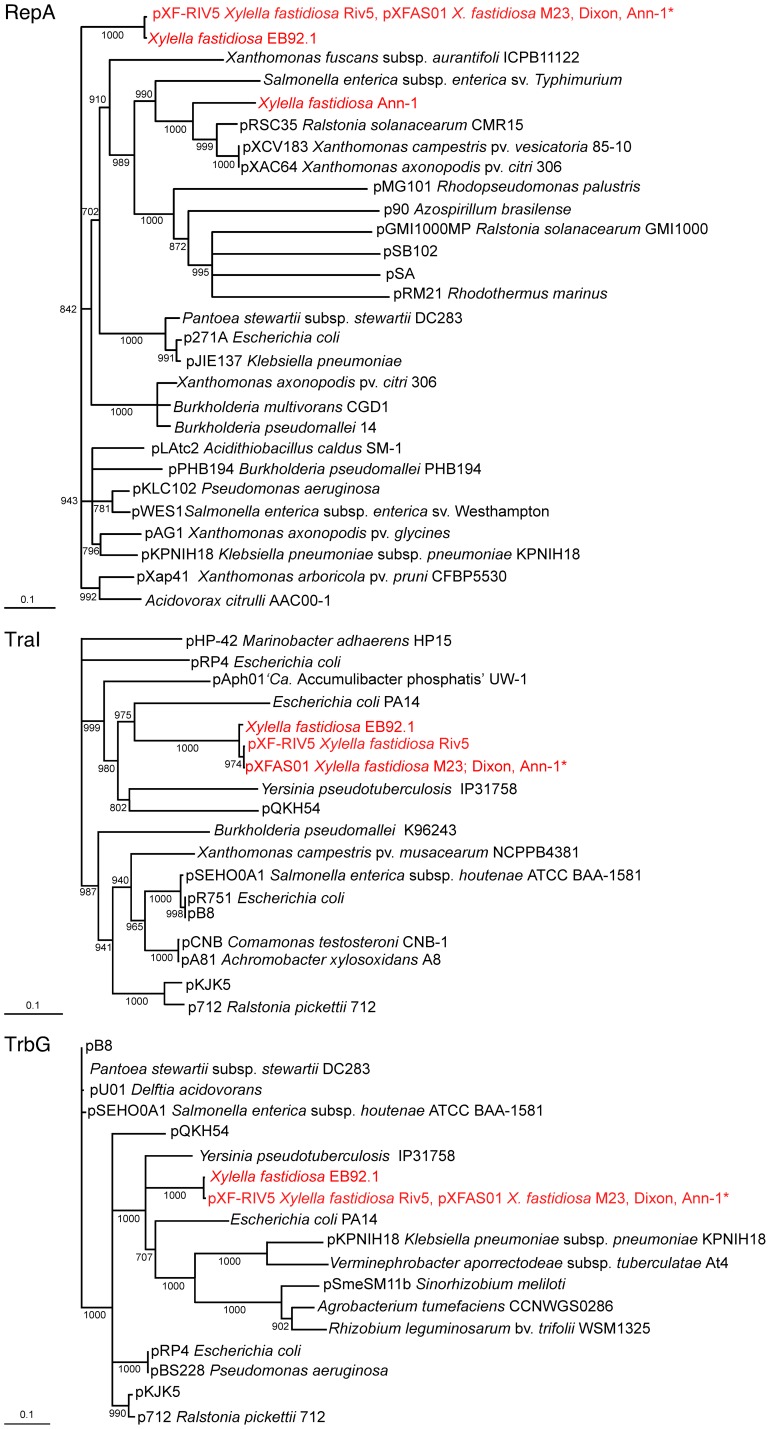
Phylogeny of RepA, TraI, and TrbG proteins encoded by pXF-RIV5. Neighbor-joining trees (1000 bootstrap replicates) presented are based on a multiple alignment of amino acid sequences. Bootstrap support values are shown for nodes with values >70%; nodes bearing ≤70% bootstrap support were collapsed to polytomies. Scale bar at lower left of each tree indicates a genetic distance of 0.1. Taxa names beginning with “p” are known to be encoded by plasmids, with plasmid designation preceding bacterial host nomenclature; replicon (plasmid versus chromosome) of other taxa is not specified. Taxa in red are encoded by strains of *Xylella fastidiosa*; multiple taxa assigned to a single branch are 100% identical. *X. fastidiosa* Ann-1 sequences identical to subspecies *multiplex* homologues are designated with an asterisk (Ann-1*) and may be derived from sequences contaminating the available Ann-1 strain genome sequence. Protein ID numbers are shown in Table S1.

Most proteins encoded by pXF-RIV5 and pXFAS01 are homologues of proteins encoded by numerous bacterial taxa. As shown in Figure 2, neighbor-joining phylogenetic trees were constructed for representative proteins (TraI and TrbG) from the two T4SS modules and for the replication protein RepA. The trees for TraI and TrbG have similar but not identical topology. The most closely related homologues (excluding those from *X. fastidiosa*) for both TraI and TrbG are from *Yersinia pseudotuberculosis* IP31758 and *E. coli* PA14. Because Tra and Trb proteins must work together to form a functional T4SS, it is not surprising that both T4SS gene clusters have similar phylogeny.

The phylogenetic history inferred for RepA is quite different from that of the T4SS (Figure 2). Most RepA homologues from *X. fastidiosa* constituted a clade distinct from RepA homologues encoded by all other taxa identified in BLAST P searches. RepA from *X. fastidiosa* strains Dixon, EB92.1 and Ann-1* were either identical, or nearly identical to that encoded by pXF-RIV5 and pXFAS01. Dixon and Riv5 are subspecies *multiplex* strains whereas M23 (host of pXFAS01) and EB92.1 are strains of subspecies *fastidiosa*. It is noted that two distinct RepA sequences are associated with the Ann-1 genome. One RepA sequence from Ann-1 (designated Ann-1*) is identical to that of pXF-RIV5, pXFAS01, and strain Dixon. The second RepA sequence from Ann-1 (designated Ann-1) shared only 53.2% amino acid sequence identity with RepA of pXF-RIV5 and pXFAS01 and clustered in a different clade with RepA from pRSC35 of *Ralstonia solanacearum* CMR15 as the most closely related homologue identified. As the Ann-1 strain genome sequence is known to be contaminated with a subspecies *multiplex* genome sequence [17], the simplest interpretation of these results is that RepA Ann-1* represents the multiplex contaminant and that RepA Ann-1 is the divergent homologue resident in the “true” Ann-1 genome of subspecies *sandyi*. Confirmation of this interpretation will require sequencing of genomes of additional strains of subspecies *sandyi*.

**Figure 3:**
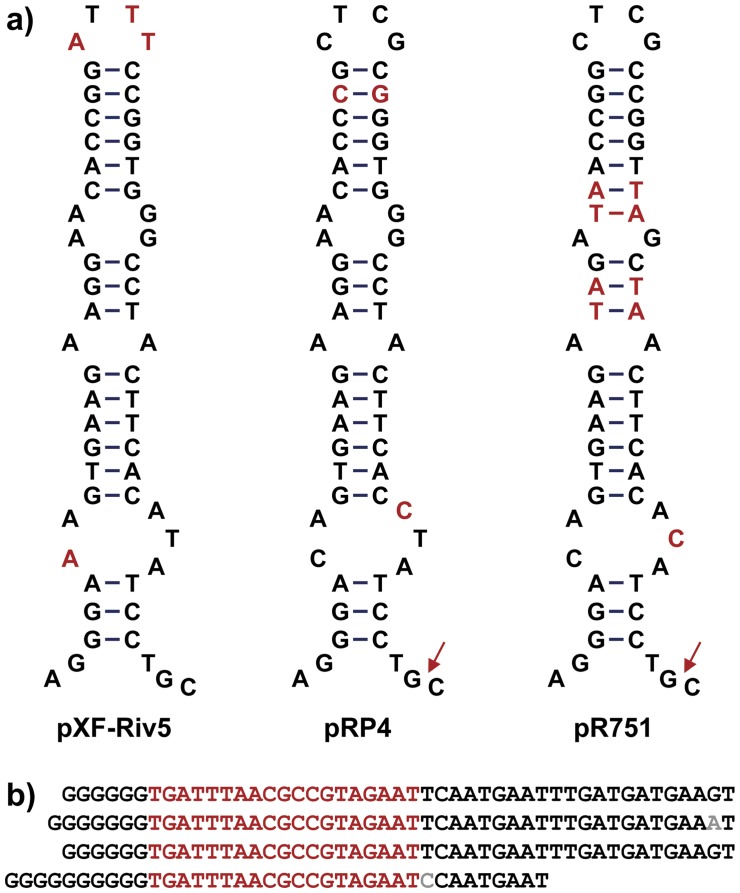
Structure of *oriT* and *oriV*. A) *oriT* inverted repeats from pXF-RIV5, pRP4, and pR751 are shown as stem-loop structures to emphasize potential base parings. Unique bases are shown in red; bases conserved in at least two structures are shown in black. Red arrows indicate experimentally determined cleavage sites in pRP4 and pR751 [21]. B) Tandem repeats in pXF-RIV5 *oriV* are aligned and the 19-bp core is shown in red. Residues that vary from the consensus are shown in gray. Nucleotides shown (34333 – 34503) are contiguous.

The presumptive origin of transfer (*oriT*) of pXF-RIV5 and pXFAS01 (nts 26869–26921) are similar to the experimentally verified *oriT* [21] from two *E. coli* plasmids (pRP4 and pR751). The stem-loop inverted repeat structure that forms the TraK binding site of pRP4 *oriT* shares 89% nucleotide sequence identity (56 of 63 nts identical) with the *oriT* homologue of pXF-RIV5 and pXFAS01 (Figure 3A). Two nucleotide substitutions in the *oriT* sequences between the *X. fastidiosa* plasmids and pRP4 are compensatory changes in the stem, preserving secondary structure (Figure 3A); the other five substitutions are in loop regions. The *oriT* region from pR751 shares less nucleotide sequence identity (79.3%; 50 of 63 bp) with pXF-RIV5 and pXF-AS01 and has one additional stem base pair relative to pXF-RIV5, pXFAS01 and pRP4.

The presumptive vegetative origin of replication (*oriV*) of pXF-RIV5 and pXFAS01 (nts 34039–34504) consists of an approximately 300 bp A+T rich region followed by four tandem repeats (Figure 3B). While the combination of an A+T rich region followed by tandem repeats is present in *oriV* of many plasmids, more common arrangements have a larger number of repeats (16 to 20) with the 19 nt core of the repeat forming a perfect 10 base pair palindrome [23]. The 19 nt core repeat sequence in pXF-RIV5 is a partial palindrome with only 6 base pairs possible.

## Discussion

As with many bacteria, *X. fastidiosa* harbors a variety of plasmids. Several have been characterized, including an IncP-1 plasmid [18], a small rolling-circle replicon [24], and pXF51 [22]. Here, we have described distinct attributes of pXF-RIV5 and pXFAS01, resident in strains representing multiple subspecies of *X. fastidiosa*. Given the probable conjugative abilities of these plasmids, it is possible that similar plasmids may be found in other as yet uncharacterized strains of *X. fastidiosa* or even in other bacterial species that share ecological niches with *X. fastidiosa*.

While many broad host range conjugative plasmids belong to the IncP-I incompatibility group and contain *trfA* homologues for vegetative replication [2], [25], replication of pXF-RIV5 and pXFAS01 uses a RepA-dependent process. Therefore, pXF-RIV5 and pXFAS01 are not readily assigned to a classic incompatibility group. Nonetheless, *tra* and *trb* genetic modules of pXF-RIV5 and pXFAS01 do cluster (Figure 2) with a subgroup typified by *E. coli* pRP4 and other IncPα group plasmids [2], [4]. It is likely that an ancestral recombination event occurred between an IncPα group plasmid similar to pRP4 and a plasmid with RepA-dependent vegetative replication to create the plasmid backbone (e.g., modules controlling replication and conjugative transfer) found in pXF-RIV5 and pXFAS01.

In addition to backbone genetic modules, many conjugative plasmids contain accessory modules encoding host-beneficial functions [26]. These accessory modules are often located at the ends of the *tra* and *trb* modules and/or near transposon or resolvase genes. No accessory modules with identifiable function were encoded by pXF-RIV5 and pXFAS01, nor by any other characterized plasmid of *X. fastidiosa*. Interestingly, ORFs for five of seven hypothetical proteins encoded by pXF-RIV5 and pXFAS01 are located downstream of both *tra* and *trb* modules, and in close proximity to a resolvase homologue (Figure 1). Whether these ORFs of unknown function constitute an accessory module conferring selective advantage to *X. fastidiosa* remains to be determined.

pXF-RIV5 and pXFAS01 are the only characterized plasmids of *X. fastidiosa* encoding all known factors (e.g., a complete T4SS) required for transfer of DNA from recipient to donor cells via conjugation. DNA transfer among strains/subspecies of *X. fastidiosa* has occurred, as evidenced by massive introgression events leading to the origin of mulberry-infecting [27] and citrus/coffee-infecting [28] strains of *X. fastidiosa*. In these cases, the mechanism of DNA transfer leading to homologous recombination appears to be transformation, as lengths of recombinant regions were generally small, albeit numerous. Indeed, recent evidence suggests that *X. fastidiosa* is naturally competent for acquisition of foreign DNA with intrinsic transformation efficiency higher than that of many other bacterial species [29]. Thus, the identification of pXF-RIV5 and pXFAS01, bearing all known hallmarks of a conjugative plasmid, suggests that *X. fastidiosa*, a plant pathogen of significant economic concern, also may transfer large segments of DNA via conjugation. Indeed, the presence of almost identical plasmids in two separate subspecies of *X. fastidiosa* (pXF-RIV5 in *multiplex* and pXFAS01 in *fastidiosa*) implies a recent inter-subspecies translocation event. Subspecies *multiplex* is relatively diverse and, therefore, likely has been present in the U. S. for a considerable time; subspecies *fastidiosa* in the U. S. exhibits limited genetic diversity. It has been hypothesized that all strains of subspecies *fastidiosa* in the U. S. are derived from a single introduction from Central America that occurred circa 1880 [8]. If so, the inter-subspecies plasmid translocation event responsible for host associations of pXF-RIV5 and pXFAS01 occurred more recently than 1880. Collectively, these observations suggest that the introduction of exotic subspecies of *X. fastidiosa* further complicates disease management, as newly introduced *X. fastidiosa* subspecies not only may cause disease(s) previously not known to occur in a region, they also provide a wealth of genetic diversity to be shared with endemic subspecies.

## Supporting Information

Table S1 **GenBank protein ID numbers for all proteins appearing in phylogenetic trees** (**Figure 2**)**.**
(DOCX)
