# Supplementary material for: A Conjugative 38 kB Plasmid Is Present in Multiple Subspecies of Xylella fastidiosa
Source: PLoS One. 2012 Dec 14;7(12):e52131. doi: 10.1371/journal.pone.0052131 (PMC3522642; doi:10.1371/journal.pone.0052131)
Supplement: Table S1 — GenBank protein ID numbers for all proteins appearing in phylogenetic trees ( Figure 2 ). (DOCX) [file pone.0052131.s001.docx]

**Supplemental Table 1**: GenBank protein ID numbers for all proteins appearing in phylogenetic trees (Figure 2).

RepA

| taxon | protein ID |
| --- | --- |
| pXF-RIV5 *Xylella fastidiosa* Riv5 | tbd |
| pXFAS01 *Xylella fastidiosa* M23 | 182682769 |
| *Xylella fastidiosa* Ann-1* | 71900107 |
| *Xylella fastidiosa* Dixon | 71276603 |
| *Xylella fastidiosa* EB92.1 | 338177745 |
| *Xanthomonas fuscans* subsp*. aurantifoli* ICPB11122 | 294624391 |
| *Salmonella enterica* subsp*. enterica* sv*. Typhimurium* | 75409916 |
| *Xylella fastidiosa* Ann-1 | 71900550 |
| pRSC35 *Ralstonia solanacearum* CMR15 | 299065053 |
| pXCV183 *Xanthomonas campestris* pv*. vesicatoria* 85-10 | 78045407 |
| pXAC64 *Xanthomonas axonopodis* pv*. citri* 306 | 21264284 |
| pMG101 *Rhodopseudomonas palustris* | 75420371 |
| p90 *Azospirillum brasilense* | 75416372 |
| pGMI1000MP *Ralstonia solanacearum* GMI1000 | 17548222 |
| pSB102 | 15919955 |
| pSA | 75346045 |
| pRM21 *Rhodothermus marinus* | 75349002 |
| *Pantoea stewartii* subsp*. stewartii* DC283 | 378579014 |
| p271A *Escherichia coli* | 342162648 |
| pJIE137 *Klebsiella pneumoniae* | 297593571 |
| *Xanthomonas axonopodis* pv*. citri* 306 | 21243174 |
| *Burkholderia multivorans* CGD1 | 221214172 |
| *Burkholderia pseudomallei* 14 | 167740614 |
| pLAtc2 *Acidithiobacillus caldus* SM-1 | 340783934 |
| pPHB194 *Burkholderia pseudomallei* PHB194 | 257123976 |
| pKLC102 *Pseudomonas aeruginosa* | 37955783 |
| pWES1 *Salmonella enterica* subsp*. enterica* sv*.*Westhampton | 216700346 |
| pAG1 *Xanthomonas axonopodis* pv. *glycines* | 190410574 |
| pKPNIH18 *Klebsiella pneumoniae* subsp. *pneumoniae* KPNIH18 | 397417397 |
| pXap41 *Xanthomonas arboricola* pv*. pruni* CFBP5530 | 351673879 |
| *Acidovorax citrulli* AAC00-1 | 120609095 |

TraI

| taxon | protein ID |
| --- | --- |
| pHP-42 *Marinobacter adhaerens* HP15 | 385334124 |
| pRP4 *Escherichia coli* | 464929 |
| pAph01 *Ca.* Accumulibacter phosphatis UW-1 | 257091574 |
| *Escherichia coli* PA14 | 390715577 |
| *Xylella fastidiosa* EB92.1 | 338177732 |
| pXF-RIV5 *Xylella fastidiosa* Riv5 | tbd |
| pXFAS01 *Xylella fastidiosa* M23 | 182682757 |
| *Xylella fastidiosa* Dixon | 71276594 |
| *Xylella fastidiosa* Ann-1* | 71900098 |
| *Yersinia pseudotuberculosis* IP31758 | 153930611 |
| pQKH54 | 108524648 |
| *Burkholderia pseudomallei* K96243 | 53720874 |
| *Xanthomonas campestris* pv*. musacearum* NCPPB4381 | 289669118 |
| pSEHO0A1 *Salmonella enterica* subsp*. houtenae* ATCC BAA-1581 | 379049192 |
| pR751 *Escherichia coli* | 464928 |
| pB8 | 77993261 |
| pCNB *Comamonas testosteroni* CNB-1 | 190572005 |
| pA81 *Achromobacter xylosoxidans* A8 | 58616682 |
| pKJK5 | 111038097 |
| p712 *Ralstonia pickettii* 712 | 388482603 |

TrbG

| taxon | protein ID |
| --- | --- |
| pB8 | 77993236 |
| *Pantoea stewartii* subsp*. stewartii* DC283 | 378582867 |
| pU01 *Delftia acidovorans* | 34500496 |
| pSEHO0A1 *Salmonella enterica* subsp*. houtenae* ATCC BAA-1581 | 379049173 |
| pQKH54 | 108524611 |
| *Yersinia pseudotuberculosis* IP31758 | 153930568 |
| *Xylella fastidiosa* EB92.1 | 338177755 |
| pXF-RIV5 *Xylella fastidiosa* Riv5 | tbd |
| pXFAS01 *Xylella fastidiosa* M23 | 182682738 |
| *Xylella fastidiosa* Dixon | 71276608 |
| *Xylella fastidiosa* Ann-1* | 71900112 |
| *Escherichia coli* PA14 | 390715592 |
| pKPNIH18 *Klebsiella pneumoniae* subsp. *pneumoniae* KPNIH18 | 397419766 |
| *Verminephrobacter aporrectodeae* subsp*. tuberculatae* At4 | 347819285 |
| pSmeSM11b *Sinorhizobium meliloti* | 190410132 |
| *Agrobacterium tumefaciens* CCNWGS0286 | 355533036 |
| *Rhizobium leguminosarum* bv*. trifolii* WSM1325 | 241518230 |
| pRP4 *Escherichia coli* | 152561 |
| pBS228 *Pseudomonas aeruginosa* | 114881127 |
| pKJK5 | 111038061 |
| p712 *Ralstonia pickettii* 712 | 388482619 |
